# Determinants of Survival Benefit From Endobiliary Radiofrequency Ablation for Extrahepatic Cholangiocarcinoma According to Tumor Stage, Systemic Therapy, and Session Number

**DOI:** 10.1055/a-2900-1143

**Published:** 2026-07-30

**Authors:** Tadahisa Inoue, Michihiro Yoshida, Naoaki Yamada, Rena Kitano, Tomoya Kitada, Masato Yano, Shun Futagami, Kenta Kachi, Fumihiro Okumura, Itaru Naitoh

**Affiliations:** 1 Department of Gastroenterology 12703 Aichi Medical UniversityNagakuteAichi PrefectureJapan; 2 Department of Gastroenterology and Metabolism 38386 Nagoya City University Graduate School of Medical Sciences and Medical SchoolNagoyaJapan; 3 Department of Gastroenterology 37079 Gifu Prefectural Tajimi HospitalTajimiGifuJapan; 4 Department of GastroenterologyNagoya City University Midori Municipal HospitalNagoyaJapan

**Keywords:** endobiliary radiofrequency ablation, extrahepatic cholangiocarcinoma, overall survival, systemic therapy

## Abstract

**Background** Endobiliary radiofrequency ablation (EB-RFA) has emerged as a promising adjunct capable of prolonging survival in patients with extrahepatic cholangiocarcinoma (eCCA), though its clinical utility remains unclear. Key unresolved issues include the impact of metastatic status, systemic therapy, and the number of ablation sessions on EB-RFA effectiveness. In this study, we aimed to address these factors to define the role and indications for EB-RFA better.

**Methods** This multicenter, retrospective study included 336 patients with unresectable eCCA who underwent endoscopic biliary stenting with ( *n* = 121) or without ( *n* = 215) EB-RFA. The primary outcome was overall survival (OS), which was analyzed separately for patients with and without distant metastases.

**Results** Among patients without distant metastases, EB-RFA significantly improved OS compared to stenting alone (median 527 vs. 300 days, *P* < 0.001), regardless of systemic therapy (with systemic therapy: 611 vs. 344 days, *P* = 0.005; without systemic therapy: 415 vs. 282 days, *P* = 0.040). Patients who received multiple EB-RFA sessions (≥2) had the longest OS (573 days), followed by those who received a single session (426 days) and those without ablation (300 days) ( *P* = 0.002). No OS benefit was observed in patients with distant metastases. However, among those receiving systemic therapy, multiple EB-RFA sessions significantly improved OS compared to non-ablation (274 vs. 163 days, *P* = 0.044). Adverse event rates were comparable between patients with and without EB-RFA.

**Conclusions** EB-RFA significantly improved the survival of patients with locoregional eCCA. In cases of metastasis, multiple sessions may offer additional benefits when combined with systemic therapy.

## Introduction

The prognosis of cholangiocarcinoma (CCA), which is classified into intrahepatic and extrahepatic CCA (eCCA) based on anatomical location, is significantly poorer than that of most malignancies. 1 Although surgical resection is currently the only curative option, many patients are diagnosed at advanced stages, making them unsuitable for surgery. Furthermore, a considerable proportion of patients are deemed unfit for surgery due to the need for highly invasive procedures, particularly in the context of advanced age or underlying comorbidities.

Therefore, systemic therapy continues to serve as a cornerstone in the management of CCA. Recently, in addition to conventional chemotherapy, immune checkpoint inhibitors have been introduced as part of combination regimens. 2 3 Nonetheless, treatment outcomes remain suboptimal, with the median overall survival (OS) still hovering at approximately 1 year. This underscores the urgent need for novel and effective therapeutic approaches for managing unresectable CCA.

Most patients with eCCA develop biliary strictures that lead to obstructive jaundice and cholangitis. Effective management of these strictures is essential to enable the initiation and continuation of systemic therapy and to relieve symptoms. Endoscopic biliary stent placement is currently recommended as the first-line intervention due to its effectiveness and minimally invasive nature. 4

In this context, there has been growing interest in endobiliary radiofrequency ablation (EB-RFA), in which direct treatment is applied to the tumor within the bile duct during endoscopic stent placement. 5 6 A recent meta-analysis 7 of randomized controlled trials (RCTs) suggested that EB-RFA may prolong OS in patients with eCCA, making it a promising adjunctive modality. However, several critical questions remain unanswered. First, it is unclear whether EB-RFA is equally effective in patients with locoregional diseases and distant metastases. Second, the potential additive benefits of combining EB-RFA with systemic therapy have not been clearly established. Third, the optimal number of ablation sessions required to achieve maximal therapeutic benefit remains unknown. These factors are likely to influence survival outcomes; however, previous RCTs have employed heterogeneous protocols, creating uncertainty in clinical decision-making and in the appropriate selection of patients for this intervention.

In this study, we aimed to address these issues through a multifactorial evaluation based on tumor stage, systemic therapy status, and number of EB-RFA sessions, with the goal of better defining the clinical role and indications for EB-RFA in patients with eCCA.

## Patients and Methods

### Study Design and Patients

This was a retrospective, multicenter, comparative study conducted at four Japanese institutions. We evaluated consecutive patients with unresectable malignant biliary obstruction due to eCCA who underwent successful endoscopic biliary stenting between 2011 and 2024. The exclusion criteria were as follows: patients younger than 18 years of age; those with surgically altered anatomy; a history of biliary reconstruction; procedures performed via non-transpapillary approaches; histological subtypes other than adenocarcinoma; patients receiving investigational or nonstandard systemic therapies; incomplete data regarding procedural details and clinical outcomes; and patients who declined participation.

The study was approved by the institutional review board of each participating center and was conducted in accordance with the Declaration of Helsinki. Given the retrospective nature of this study, an opt-out option was used, allowing patients to decline the use of their data for research purposes.

### Procedure

A standard duodenoscope (Olympus Medical Systems, Tokyo, Japan) was advanced into the duodenum, and biliary cannulation was performed using a standard catheter and either a 0.025- or 0.035-in. guidewire. After advancing the guidewire through the stricture into the target intrahepatic bile duct, endoscopic sphincterotomy was performed in all patients. Subsequently, either plastic or metal stents were conventionally placed in patients who were not scheduled to undergo EB-RFA.

In patients scheduled for EB-RFA, either a Habib EndoHPB catheter (Boston Scientific, Marlborough, MA, USA) or an ELRA catheter (STARmed, Goyang, South Korea) was inserted over the guidewire. For the Habib catheter, the stricture was ablated for 90 s at a power of 7 or 10 W using a VIO300D generator (ERBE Elektromedizin GmbH, Tübingen, Germany). For the ELRA catheter, ablation was performed for 120 s at a power of 7 or 10 W with a target temperature of 80 °C, using a VIVA combo generator (STARmed). If the stricture length exceeded the ablation range of a single application or involved both the right and left hepatic ducts, ablation was repeated sequentially to cover the entire stricture length. After ablation, balloon cleaning of the bile duct was performed, followed by the placement of plastic or metal stents to cover the treated stricture. The choice between plastic stents and metal stents was determined at the discretion of the treating endoscopist. Plastic stents were mainly 7-Fr stents, whereas metal stents were mainly 8- or 10-mm self-expandable metal stents. For hilar biliary strictures, bilateral drainage was generally attempted as the standard strategy.

Additionally, in some patients, scheduled reablation was performed at intervals of approximately 3 months or at the time of stent occlusion. In such cases, the plastic stent was removed, EB-RFA was repeated at the stricture site using the same settings as in the initial session, and a new stent was then inserted.

All procedures were performed by or under the direct supervision of experienced endoscopists with expertise in therapeutic endoscopic retrograde cholangiopancreatography.

### Outcomes and Definitions

The primary outcome of this study was OS. Secondary outcomes included adverse events (AEs) associated with the procedure. The study population was categorized into two groups: patients who underwent EB-RFA, followed by stenting (RFA group) and those who underwent stenting alone (non-RFA group). The outcomes were compared between the two groups. The primary outcome, OS, was analyzed separately in two cohorts: patients without distant metastases and those with distant metastases. Additionally, subgroup analyses were conducted based on systemic therapy status and the number of EB-RFA sessions (single-session vs. multiple-session EB-RFA).

OS was defined as the interval from the date of initial stenting (with or without EB-RFA) to the date of death. AEs were defined and graded according to the criteria provided by the American Society for Gastrointestinal Endoscopy. 8 AEs occurring within 14 days of the initial procedure were classified as early AE, while those occurring after 14 days were classified as late AE. Recurrent biliary obstruction (RBO) was defined as the recurrence of jaundice or cholangitis with biliary dilation on imaging studies or any other clinical condition necessitating additional biliary drainage or stent removal. 9 Scheduled stent exchanges, including those performed in conjunction with repeated ablation sessions, were not considered RBO events.

### Statistical Analysis

Categorical variables were compared using Fisher’s exact test, while continuous variables were analyzed using the Mann–Whitney *U* test. OS was evaluated using the Kaplan–Meier method, and survival curves were compared using the log-rank test. Data were censored for patients who were alive at the time of study termination or transferred to another institution, including hospice care, when further follow-up was no longer possible.

All statistical analyses were performed using EZR version 1.68 (Saitama Medical Center, Jichi Medical University, Saitama, Japan). 10 A *P* -value of <0.05 was considered statistically significant.

## Results

### Patients

A total of 336 patients met the inclusion criteria: 121 in the RFA group and 215 in the non-RFA group. Among them, 166 patients were included in the cohort without distant metastases (41 in the RFA group and 125 in the non-RFA group), and 170 patients were included in the cohort with distant metastases (80 in the RFA group and 90 in the non-RFA group). No significant differences were observed in baseline patient characteristics between the RFA and non-RFA groups in either cohort ( Table 1 ). Among the 121 patients who underwent EB-RFA, the Habib EndoHPB catheter was used in 106 patients, whereas the ELRA catheter was used in 15 patients.

**Table 1:** Baseline characteristics of the patients.

	All ( *n* = 336)	Without distant metastasis			With distant metastasis		
		RFA group ( *n* = 41)	Non-RFA group ( *n* = 125)	*P* value	RFA group ( *n* = 80)	Non-RFA group ( *n* = 90)	*P* value
Sex, (male/female), *n*	205/131	26/15	83/42	0.850	49/31	47/43	0.279
Age, years		83 (77–86)	83 (79–87)	0.625	77 (68–83)	72 (66–81)	0.117
Performance status, *n* (%)				0.738			0.649
≤2	301 (89.6)	37 (90.2)	116 (92.8)		71 (88.8)	77 (85.6)	
≥3	35 (10.4)	4 (9.8)	9 (7.2)		9 (11.2)	13 (14.4)	
Tumor location, *n* (%)				0.359			0.865
Hilar							
Bismuth type ≤III	169 (50.3)	22 (53.7)	52 (41.6)		46 (57.5)	49 (54.4)	
Bismuth type IV	94 (28.0)	10 (24.4)	33 (26.4)		24 (30.0)	27 (30.0)	
Distal	73 (21.7)	9 (22.0)	40 (32.0)		10 (12.5)	14 (15.6)	
Stricture length, mm, median (IQR)		23 (18–25)	26 (19–34)	0.244	22 (15–28)	20 (18–34)	0.148
Jaundice, ^†^ *n* (%)	284 (84.5)	34 (82.9)	101 (80.8)	1.000	66 (82.5)	83 (92.2)	0.064
Cholangitis, ^†^ *n* (%)	51 (15.2)	15 (29.4)	24 (19.2)	0.163	5 (6.2)	7 (7.8)	0.771
Type of initial stent, *n* (%)				0.563			0.107
Metal stent	242 (72.0)	26 (63.4)	87 (69.6)		56 (70.0)	73 (81.1)	
Plastic stent	94 (28.0)	15 (36.6)	38 (30.4)		24 (30.0)	17 (18.9)	
Number of stents placed, *n* (%)				0.323			0.730
1	93 (27.7)	9 (22.0)	39 (31.2)		20 (25.0)	25 (27.8)	
≥2	243 (72.3)	32 (31.2)	86 (68.8)		60 (75.0)	65 (72.2)	
Systemic therapy, *n* (%)	165 (49.1)	18 (43.9)	35 (28.0)	0.081	54 (67.5)	58 (64.4)	0.747
GC	93	14	14		29	36	
GCD/GCP	42	4	10		14	14	
GEM	16	0	7		1	8	
S-1	14	0	4		10	0	
EB-RFA, *n* (%)	121 (36.0)			–			–
Single session	82	26	–		56	–	
Multiple session	39	15	–		24	–	

RFA: radiofrequency ablation; IQR: interquartile range; GC: gemcitabine + cisplatin; GCD: gemcitabine + cisplatin + durvalumab; GCP: gemcitabine + cisplatin + pembrolizumab; GEM: gemcitabine; EB-RFA: endobiliary radiofrequency ablation.

^†^ Before biliary drainage.

### OS in patients without distant metastasis

**Fig. 1:**
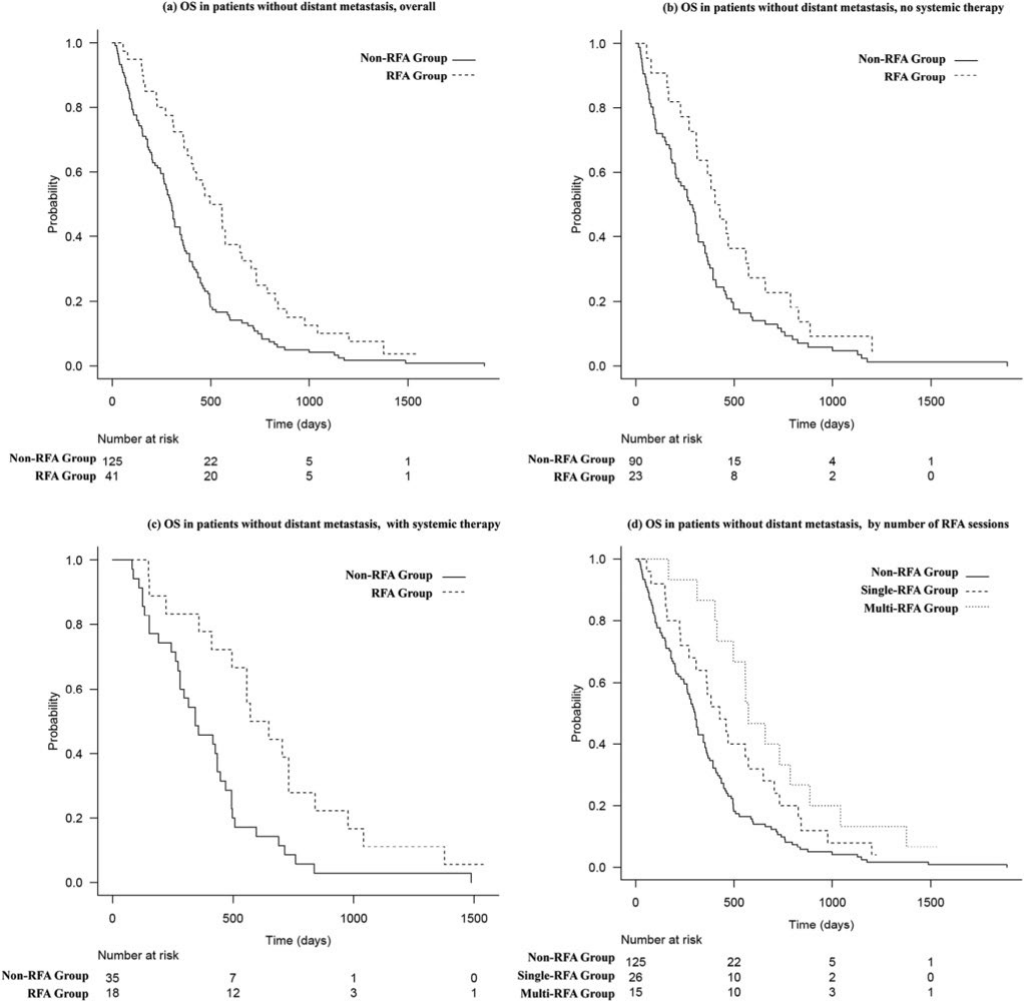
Kaplan–Meier analysis of OS in patients with eCCA without distant metastasis. ( **a** ) OS in the overall cohort without distant metastasis. Median OS was significantly longer in the RFA group than in the non-RFA group (527 vs. 300 days, *P* < 0.001). ( **b** ) OS in patients not receiving systemic therapy. The RFA group showed a significantly longer OS than the non-RFA group (415 vs. 282 days, *P* = 0.040). ( **c** ) OS in patients receiving systemic therapy. Median OS was significantly longer in the RFA group than in the non-RFA group (611 vs. 344 days, *P* = 0.005). ( **d** ) OS stratified by the number of RFA sessions. Patients who underwent multiple RFA sessions (≥2) had the longest OS (573 days), followed by those with a single session (426 days), and the non-RFA group (300 days) ( *P* = 0.002). OS: overall survival; eCCA: extrahepatic cholangiocarcinoma; RFA: radiofrequency ablation.

In the cohort without distant metastasis, the median OS was significantly longer in the RFA group than in the non-RFA group: 527 days (95% confidence interval [CI], 382–649 days) versus 300 days (95% CI, 260–344 days), respectively ( *P* < 0.001) ( Fig. 1a ).

Subgroup analysis of patients who did not receive systemic therapy showed a similar trend. The RFA group showed significantly prolonged survival compared to the non-RFA group: 415 days (95% CI, 270–573 days) versus 282 days (95% CI, 202–317 days) ( *P* = 0.040) ( Fig. 1b ). In patients receiving systemic therapy, the median OS was also significantly longer in the RFA group than in the non-RFA group: 611 days (95% CI, 412–731 days) versus 344 days (95% CI, 272–435 days), respectively ( *P* = 0.005) ( Fig. 1c ).

A stratified analysis based on the number of EB-RFA sessions revealed a significant difference, with the longest median OS observed in patients who underwent multiple sessions (≥2 sessions; 573 days, 95% CI, 403–786 days), followed by those who received a single session (426 days; 95% CI, 270–649 days) and the non-RFA group (300 days; 95% CI, 260–344 days) ( *P* = 0.002) ( Fig. 1d ). Among patients in the multiple-session group, 93.3% (14/15) underwent two sessions, and 6.7% (1/15) underwent three or more sessions. The median interval between EB-RFA sessions was 3 months (interquartile range, 2.5–3 months).

### OS in patients with distant metastasis

**Fig. 2:**
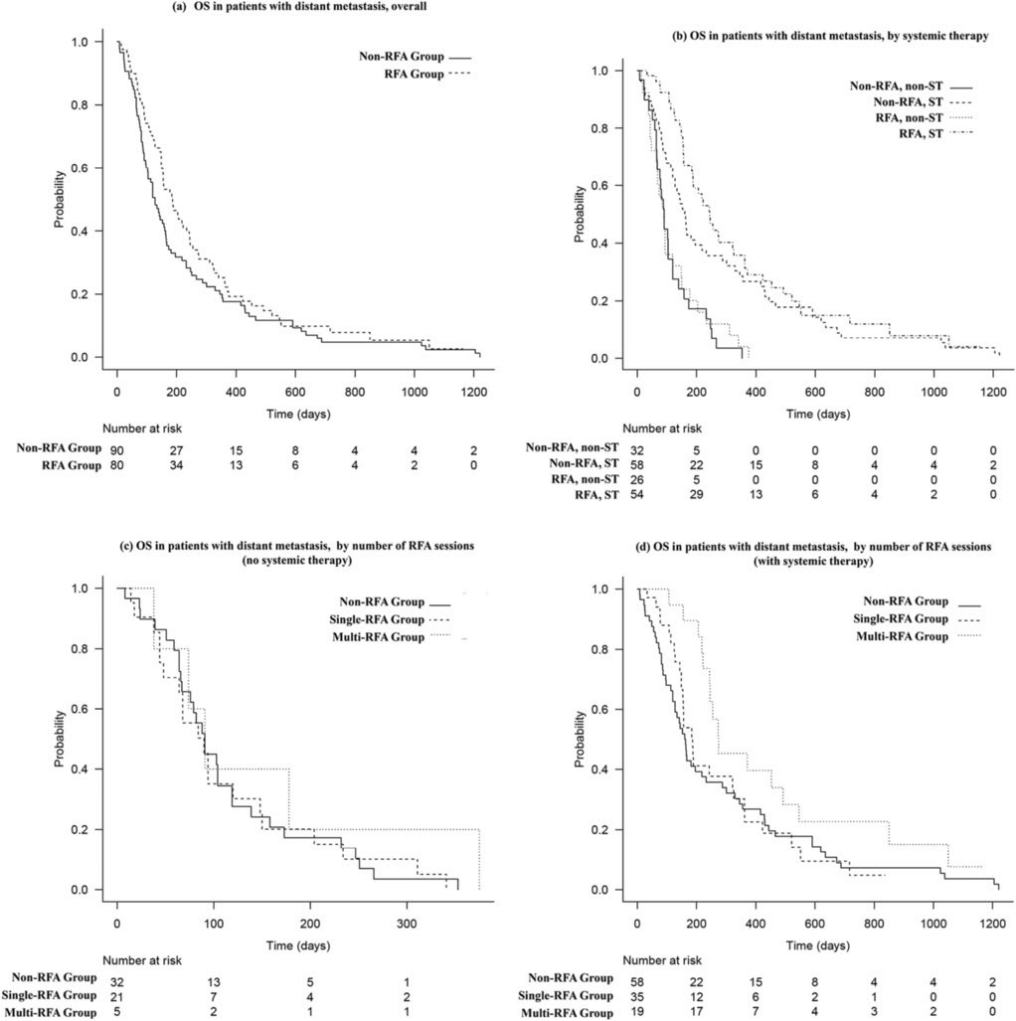
Kaplan–Meier analysis of OS in patients with eCCA and distant metastasis. ( **a** ) OS in the overall cohort with distant metastasis. There was no significant difference in OS between the RFA group and the non-RFA group (median 187 vs. 127 days, *P* = 0.136). ( **b** ) OS by systemic therapy status. Among patients not receiving systemic therapy, no significant difference in OS was observed between the RFA and non-RFA groups (90 vs. 91 days, *P* = 0.750). Similarly, among patients who received systemic therapy, the difference in OS between the RFA and non-RFA groups (244 vs. 163 days) was not statistically significant ( *P* = 0.153). ( **c** ) OS in patients not receiving systemic therapy, stratified by the number of RFA sessions. No significant differences in OS were observed between the multiple-session RFA (median 91 days), the single-session RFA (90 days), and the non-RFA groups (91 days) ( *P* = 0.882). ( **d** ) OS in patients receiving systemic therapy, stratified by the number of RFA sessions. The multisession RFA group had the longest median OS (274 days), followed by the single-session RFA group (187 days) and the non-RFA group (163 days). Compared with the non-RFA group, the single-session RFA group did not show a statistically significant difference ( *P* = 0.696), whereas the multisession RFA group showed a significant survival benefit ( *P* = 0.044). OS: overall survival; eCCA: extrahepatic cholangiocarcinoma; RFA: radiofrequency ablation.

In the cohort with distant metastases, the median OS was 187 days (95% CI, 148–243 days) in the RFA group and 127 days (95% CI, 97–163 days) in the non-RFA group; however, this difference was not statistically significant ( *P* = 0.136) ( Fig. 2a ).

Among patients who did not receive systemic therapy, the median OS was nearly identical between the RFA (90 days; 95% CI, 64–148 days) and non-RFA groups (91 days; 95% CI, 66–119 days), with no significant difference observed ( *P* = 0.750) ( Fig. 2b ). In patients who received systemic therapy, the median OS was also not statistically different between the RFA (244 days; 95% CI, 187–325 days) and non-RFA groups (163 days; 95% CI, 119–218 days) ( *P* = 0.153) ( Fig. 2b ).

In a stratified analysis based on the number of EB-RFA sessions among patients not receiving systemic therapy, no significant differences in OS were observed between the multiple-session RFA (91 days; 95% CI, 38–NA), single-session RFA (90 days; 95% CI, 48–148 days), and non-RFA groups (91 days; 95% CI, 66–119 days) ( *P* = 0.882) ( Fig. 2c ).

Among the patients who received systemic therapy, the median OS was the longest in the multisession RFA group (274 days; 95% CI, 222–492 days), followed by the single-session RFA group (187 days; 95% CI, 148–325 days) and the non-RFA group (163 days; 95% CI, 119–218 days). Compared with the non-RFA group, the single-session RFA group did not show a statistically significant difference ( *P* = 0.696), while the multisession RFA group showed a significant survival benefit ( *P* = 0.044) ( Fig. 2d ).

Among the patients who underwent multisession RFA, 79.2% (19/24) underwent two sessions, and 20.8% (5/24) underwent three or more sessions. The median interval between RFA sessions was 3 months (interquartile range, 2–3 months).

### AEs

AEs other than RBO occurred in 19.8% (24/121) of the patients in the RFA group and 16.3% (35/215) in the non-RFA group, with no significant difference between the groups ( *P* = 0.456) ( Table 2 ). Early AEs (≤14 days) were observed in 6.6% (8/121) and 10.7% (23/215) of patients, respectively ( *P* = 0.244). Most early AEs were of mild-to-moderate severity, and severe events were reported only in the non-RFA group.

**Table 2:** Comparison of adverse events between the radiofrequency ablation and non-radiofrequency ablation groups.

	RFA group ( *n* = 121)	Non-RFA group ( *n* = 215)	*P* value
Patients with any AE besides RBO, *n* (%)	24 (19.8)	35 (16.3)	0.456
Early AE besides RBO (≤14 days after initial procedure), *n* (%)	8 (6.6)	23 (10.7)	0.244
Cholecystitis	2	4	
Hemobilia	2	1	
Aspiration pneumonia	2	0	
Cholangitis	1	4	
Pancreatitis	1	10	
Liver abscess	0	2	
Post-EST bleeding	0	1	
Cardiovascular event	0	1	
Severity of early AE, *n* (%)			
Mild	2	12	
Moderate	6	9	
Severe	0	2	
Late AE besides RBO (≥15 days after initial procedure), ^†^ *n* (%)	16 (13.2)	16 ^‡^ (7.4)	0.120
Cholangitis	8	2	
Cholecystitis	4	2	
Liver abscess	4	12	
Severity of late AE, *n* (%)			
Mild	0	0	
Moderate	16	16	
Severe	0	0	
RBO, ^§^ *n* (%)	52 (43.0)	115 (53.5)	0.070

RFA: radiofrequency ablation; RBO: recurrent biliary obstruction; AE: adverse event; EST: endoscopic sphincterotomy.

^†^ Late AEs include events that occurred during scheduled stent exchange including second or subsequent ablation sessions.

^‡^ Four patients may have had overlapping early and late AEs.

^§^ Scheduled stent exchanges, including those for repeat ablation sessions, were not considered as RBO.

Late AEs (≥15 days), including events during scheduled stent exchange with or without repeated ablation, were reported in 13.2% (16/121) of the RFA group and 7.4% (16/215) of the non-RFA group ( *P* = 0.120), with all cases graded as moderate.

RBO occurred in 43.0% (52/121) of patients in the RFA group and in 53.5% (115/215) of patients in the non-RFA group, with no statistically significant difference ( *P* = 0.070).

## Discussion

This study demonstrated that EB-RFA improves the OS of patients with locoregional eCCA. This survival benefit was observed regardless of the administration of systemic therapy. Although a single ablation session was associated with a survival advantage, multiple sessions appeared to provide greater benefit. In contrast, no survival benefit was observed in patients with distant metastases, irrespective of the systemic therapy status or the number of ablation sessions, except in those who underwent multiple sessions in combination with systemic therapy, in whom a significant survival benefit was evident. The incidence of AE did not increase with the use of EB-RFA.

Ablative therapies are well-established treatment modalities for various malignancies. For instance, in hepatocellular carcinoma, RFA is recommended as the first-line treatment alongside surgery, depending on the tumor size and number. In eCCA, EB-RFA is typically performed using bipolar catheters equipped with two or four electrodes at the tip. Although EB-RFA was originally developed to prolong stent patency, its efficacy remains controversial due to inconsistent findings across various RCTs and meta-analyses. 11 12 13 14 15 However, several studies, including RCTs, have suggested that EB-RFA can provide survival benefits to patients with eCCA. 16 A recent meta-analysis 7 of nine RCTs comprising 750 patients with malignant biliary obstruction reported a significant survival advantage for EB-RFA combined with stenting compared to stenting alone in patients with eCCA (mean difference: 4.9 months; 95% CI: 4.6–5.1 months; *P* < 0.001).

Despite these findings, EB-RFA remains a developing treatment modality, as key clinical questions remain unanswered. One of the key unresolved issues is the impact of tumor stage on the effectiveness of EB-RFA. Most previous RCTs did not differentiate between locoregional and metastatic diseases, leaving uncertainty about whether treatment exerts similar effects across different stages. Although the abscopal effect is a theoretical possibility, it remains unclear whether ablating only the intraductal and periductal regions is sufficient to impact the prognosis in patients with distant metastases whose outcomes are largely determined by systemic disease burden. A retrospective study by Xia et al., 17 involving 883 patients with malignant biliary obstruction of various etiologies, suggested that EB-RFA was associated with improved survival only in patients with eCCA, particularly in patients without distant metastases.

The mechanism by which EB-RFA prolongs survival likely involves both local tumor control through coagulative necrosis and the activation of antitumor immunity. 18 The release of tumor-associated antigens following ablation may stimulate immune responses via antigen presentation. Several recent studies have suggested a synergistic effect of RFA and systemic therapies, particularly immunotherapy. 19 20 In our study, the patients with metastatic disease did not benefit from EB-RFA alone. However, patients who underwent multiple ablation sessions in combination with systemic therapy showed significantly improved survival. These findings suggest that while EB-RFA alone may have a limited impact on survival in the presence of distant metastases, it may become clinically relevant when systemic therapy effectively controls metastatic lesions. Furthermore, this finding supports the hypothesis that ablation enhances tumor antigen release and may trigger immune-mediated responses capable of targeting distant metastases. This represents a potentially important benefit of EB-RFA and warrants further investigation.

Another unresolved issue is the optimal number of EB-RFA sessions required to achieve certain benefit. Previous studies have included both single and multiple EB-RFA protocols with no clear consensus. To our knowledge, our study is the first to evaluate the impact of the number of sessions on clinical outcomes. In patients without distant metastases, even one session was effective; however, multiple sessions yielded superior results. In metastatic cases, survival benefits were observed only in patients who received multiple sessions along with systemic therapy. Collectively, these findings suggest that repeated EB-RFA sessions are preferable across different disease settings. Several factors could explain this phenomenon. First, repeated ablation may enhance antigen release, potentially amplifying the antitumor immune response. Second, the current bipolar catheter design has limitations in ablating many strictures, including short or soft ones due to difficulty in achieving adequate electrode contact. 21 22 In such cases, a single session may result in suboptimal ablation, and repeated sessions may compensate for this limitation. New devices such as balloon-based ablation catheters 23 have been under development to ensure better and more consistent tissue contact, potentially improving treatment efficacy in future applications.

This study has some limitations. First, this was a retrospective, nonrandomized study, and treatment allocation to EB-RFA was determined at the discretion of the treating physicians. Therefore, selection bias and confounding by indication cannot be excluded. In particular, the absence of multivariable adjustment limits our ability to determine whether EB-RFA was independently associated with improved survival. Second, the analysis according to the number of EB-RFA sessions may have been affected by immortal time bias, because patients in the multiple-session group necessarily had to survive long enough to undergo subsequent ablation. This may have preferentially allocated early deaths to the single-session or non-RFA groups and consequently overestimated the apparent survival benefit of repeated EB-RFA. Third, several subgroup analyses, particularly those evaluating multiple EB-RFA sessions, included a limited number of patients. Therefore, these results may be statistically unstable and should be interpreted cautiously. Additionally, some patients had censored data due to transfer to other institutions or study termination, which could have influenced long-term outcomes such as OS duration. Finally, the study spanned a long period during which treatment strategies for malignant biliary obstruction and systemic therapy regimens evolved. Therefore, our findings should be interpreted as exploratory and hypothesis-generating and should be validated in well-designed prospective studies.

Nonetheless, in conclusion, this is the first study to evaluate the clinical effectiveness of EB-RFA in eCCA based on tumor stage, systemic therapy status, and number of sessions. These findings demonstrate significant survival differences associated with these factors and offer valuable evidence to guide patient selection and optimize EB-RFA implementation. Furthermore, these findings may serve as a foundation for future prospective trials.
